# Aortic Valve Calcification Across Aortic Stenosis Subtypes According to Gradient and Flow: Insights from a Single-Center Transcatheter Aortic Valve Implantation Cohort with 2-Year Mortality Outcomes

**DOI:** 10.3390/jcm15114290

**Published:** 2026-06-01

**Authors:** Karim El Zahab, Marsela Gega, Isabell Singerer, Younes Najim, Biljana Vokic, Ralf Degenhardt, Osama Bisht, Marcus Franz, Mohammad Elgarhy

**Affiliations:** 1 Department of Cardiology, Angiology and Intensive Care Medicine, Klinikum Bad Hersfeld GmbH, 36251 Bad Hersfeld, Germany; karim.el-zahab@klinikum-hef.de; 2 Department of Cardiology, Angiology and Intensive Care Medicine, Cardiovascular Center Hersfeld-Rotenburg, 36199 Rotenburg an der Fulda, Germany; m.gega@hkz-rotenburg.de (M.G.); i.singerer@hkz-rotenburg.de (I.S.); y.najim@hkz-rotenburg.de (Y.N.); b.vokic@hkz-rotenburg.de (B.V.); r.degenhardt@hkz-rotenburg.de (R.D.); m.el-garhy@hkz-rotenburg.de (M.E.); 3 Evangelisches Herzzentrum, 06869 Coswig, Germany; obisht@gmail.com; 4 Department of Cardiothoracic Surgery, University Hospital Jena, 07743 Jena, Germany; 5 Department of Internal Medicine I, University Hospital Jena, 07743 Jena, Germany

**Keywords:** aortic stenosis, transcatheter aortic valve implantation, aortic valve calcium score, low-gradient aortic stenosis

## Abstract

**Background:** The extent and distribution of valvular calcification may differ across hemodynamic subtypes of severe aortic stenosis (AS), but their clinical relevance in patients undergoing transcatheter aortic valve implantation (TAVI) remains incompletely understood. **Methods:** We retrospectively analyzed 315 consecutive patients undergoing transfemoral TAVI for symptomatic severe AS at a single center between January 2020 and December 2022. Aortic valve calcification was assessed by aortic valve calcium score (AVCS) on non-contrast CT and by calcification volume (CV) on contrast-enhanced CT. Patients were classified as high-gradient aortic stenosis (HGAS), classical low-flow low-gradient aortic stenosis (cLFLGAS), paradoxical low-flow low-gradient aortic stenosis (pLFLGAS), or normal-flow low-gradient aortic stenosis (NFLGAS). **Results:** HGAS represented 70.8% of the cohort, whereas low-gradient AS subtypes accounted for the remaining cases. Valvular calcification burden was highest in HGAS and consistently lower in all low-gradient phenotypes, particularly in pLFLGAS. The non-coronary cusp was the most heavily calcified cusp across all groups. Neither AVCS nor CV was associated with all-cause mortality up to 2 years after TAVI. **Conclusions:** Low-gradient AS subtypes exhibit a lower valvular calcification burden than HGAS, but these differences did not translate into differences in 2-year mortality after transfemoral TAVI in this particular cohort.

## 1. Introduction

High-gradient aortic stenosis (HGAS) is the most common subtype of severe AS in transcatheter aortic valve implantation (TAVI) patients. Among non-high-grade severe AS patients, three subtypes can be distinguished: classical low-flow low-gradient AS (cLFLGAS), paradoxical low-flow low-gradient AS (pLFLGAS), and normal-flow low-gradient AS (NFLGAS). The incidence of these low-gradient severe AS subtypes (LGAS subtypes) varied between 23.5 and 30% in TAVI registries [1,2]. Computed tomography (CT)-based aortic valve calcium score (AVCS) is a valuable adjunct for assessing AS severity, particularly when echocardiographic parameters are inconclusive. Thresholds for severe AS (e.g., >2000 Agatston units [AU] in men, >1200 AU in women) are suggested in guidelines to support the diagnosis of severe AS [3]. However, these thresholds are mainly obtained from severe HGAS and their diagnostic accuracy in LGAS subtypes remains limited [4]. Notably, Clavel et al. reported that only 50% of patients with LGAS subtypes exceeded these AVCS thresholds, highlighting the lower calcium burden often observed in these patients [5]. There are many factors contributing to the cumulative amount of calcification, such as the size of the annulus and calcification symmetry [6].

The objective of this study was to compare aortic valve calcification, assessed by CT-based AVCS and CV, between different AS subtypes (HGAS, cLFLGAS, pLFLGAS and NFLGAS), and to evaluate their association with 2-year all-cause mortality among patients undergoing transfemoral (tf-) TAVI for severe AS.

## 2. Methods

### 2.1. Study Design and Population

We included 315 consecutive patients who underwent tf-TAVI at the Cardiovascular Center Rotenburg (Herz-Kreislauf-Zentrum Rotenburg a.d. Fulda, Klinikum Hersfeld-Rotenburg GmbH (Bad Hersfeld, Germany)) between January 2020 and December 2022 for symptomatic severe AS, defined as an aortic valve area (AVA) < 1.0 cm^2^.

**Figure 1:**
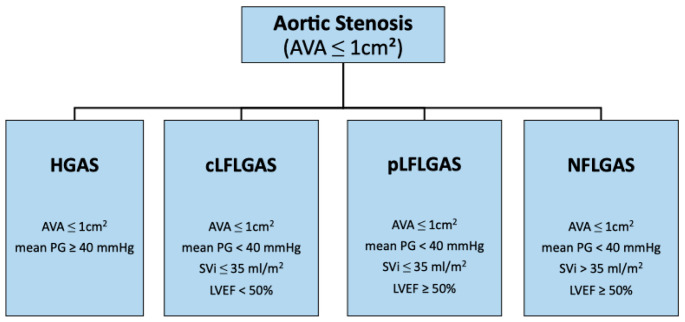
Definition of different hemodynamic subtypes of aortic stenosis as used in this study.

Patients who had undergone valve-in-valve TAVI (*n* = 11) or non-transfemoral TAVI (*n* = 9) were excluded. The final study population was stratified according to mean transaortic pressure gradient (mPG), stroke volume index (SVi), and left ventricular ejection fraction (LVEF) into four hemodynamic AS subtypes (Figure 1): HGAS, cLFLGAS, pLFLGAS, and NFLGAS. Subtype definitions were based on standard thresholds. HGAS was defined as mPG ≥ 40 mmHg. LGAS subtypes were defined as mPG < 40 mmHg and further subdivided by flow and LVEF into cLFLGAS (SVi ≤ 35 mL/m^2^ and LVEF < 50%), pLFLGAS (SVi ≤ 35 mL/m^2^ and LVEF ≥ 50%), and NFLGAS (SVi > 35 mL/m^2^ and LVEF ≥ 50%). In LGAS subtypes, AS severity was adjudicated using an integrative approach including clinical context, Doppler measures (e.g., Doppler velocity index), and, where indicated, dobutamine stress echocardiography (DSE) and/or sex-specific CT-AVCS thresholds. Since a relevant number of patients received such additional diagnostic approaches in referring clinics and outpatient departments, we cannot give exact data on the proportion of patients undergoing the different methods.

### 2.2. Ethics Approval and Consent to Participate

The study was conducted in accordance with the Declaration of Helsinki and was approved by the Ethics Committee of the State Medical Association of Hesse (Ethik-Kommission der Landesärztekammer Hessen; approval code: 2024-3857-evBO, approval date: 8 October 2024). Given the retrospective design and use of routinely collected, anonymized clinical data, the requirement for written informed consent was waived.

### 2.3. Echocardiographic Assessment

Transthoracic echocardiography (TTE) served as the primary diagnostic modality for evaluating AS. Peak and mean gradients were measured in multiple acoustic windows: apical five-chamber, long axis, right parasternal, and suprasternal views. Aortic valve area (AVA) was calculated using the continuity equation. Left ventricular ejection fraction (LVEF) was assessed via the biplane Simpson method. Stroke volume was calculated at the left ventricular outflow tract and indexed to body surface area. DSE was selectively performed in patients with low-gradient AS and LVEF < 50% [3].

### 2.4. Pre-Procedural Computed Tomography (CT) Imaging and Calcium Analysis

All patients underwent multislice CT (MSCT) angiography for TAVI planning, acquired with a Siemens Somatom Definition Edge scanner. Images were obtained using a retrospective ECG-gated protocol with 70 mL of iopamidol (Ultravist-370, Bayer, Leverkusen, Germany). Acquisition parameters were tailored to the patient’s body habitus. All measurements were performed by 2 independent experienced cardiac imaging specialists blinded for both hemodynamic AS subtype and outcome. The aortic valve calcium score (AVCS) was quantified using non-contrast CT (ncCT) with a threshold of 130 Hounsfield units (HU). The calcification volume (CV) was measured using contrast-enhanced CT (ceCT) with a modifiable threshold. To ensure consistency, we used a standardized approach starting with a 450 HU threshold—commonly accepted for differentiating calcium from contrast-enhanced blood in TAVI planning CT—and manually adjusted it only when necessary, based on visual confirmation of true calcification. Borderline cases were independently reviewed by two observers, with disagreements resolved by consensus together with the senior author. Although formal interobserver variability analysis was not performed, this structured dual-review process ensured reproducibility and minimized subjective variability.

### 2.5. Mortality and Outcomes

All-cause mortality was assessed at 30 days, 1 year and 2 years after TAVI. Survival was analyzed using the Kaplan–Meier method, with comparisons between subgroups performed using the log-rank test.

Periprocedural complications (within 30 days) were extracted from institutional records and included vascular complications, bleeding events (overall and bleeding requiring transfusion), pericardial tamponade, new permanent pacemaker implantation, and in-hospital mortality. Where sufficient information was available, bleeding and vascular complications were classified according to VARC-3 definitions. Follow-up for mortality was obtained from hospital records and contact with referring physicians; only all-cause mortality was analyzed.

### 2.6. Statistical Analysis

Continuous variables were tested for normal distribution using the Shapiro–Wilk test. Parametric data are presented as mean ± standard deviation and non-parametric data as median with interquartile range (IQR). Comparisons across the four hemodynamic AS subtypes were performed using one-way ANOVA (with Tukey post hoc testing) for normally distributed variables or the Kruskal–Wallis test (with Dunn–Bonferroni post hoc testing) for non-normally distributed variables. Categorical variables were compared using the chi-square test or Fisher’s exact test, as appropriate. All-cause mortality was analyzed using Kaplan–Meier curves with log-rank testing. Given the very limited (too low) number of outcome events in some subgroups, mortality analyses were considered exploratory and were not adjusted for covariates. All analyses were performed using SPSS software (version 24.0, IBM Corp., Armonk, NY, USA). A two-tailed *p*-value < 0.05 was considered statistically significant.

## 3. Results

### 3.1. Patient Cohort and Baseline Characteristics

**Figure 2:**
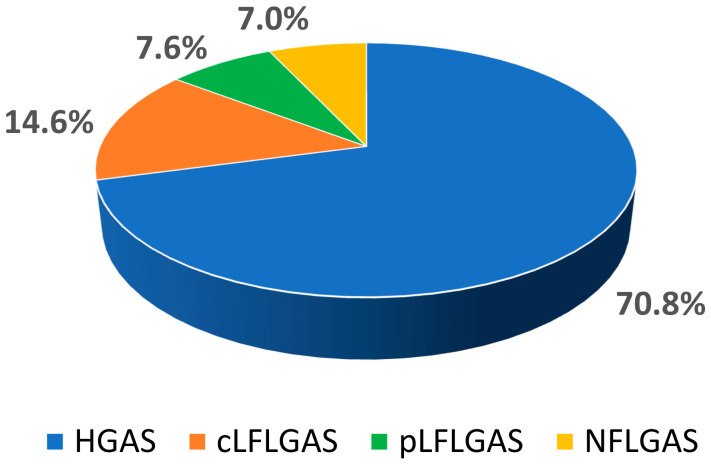
Incidence of AS subtypes across the study cohort.

The study cohort comprised 315 patients undergoing tf-TAVI for severe AS. HGAS represented 70.8% of cases, whereas cLFLGAS, pLFLGAS, and NFLGAS accounted for 14.6%, 7.6%, and 7.0%, respectively (Figure 2). Across subtypes, mean age ranged from 81.4 to 81.9 years without meaningful differences. Body mass index (BMI) was similar across groups (overall 27.3 ± 4.8 kg/m^2^). The proportion of female patients was highest in the HGAS group (53.8%) and lowest in the NFLGAS (40.9%) and cLFLGAS (39.1%) groups. Coronary artery disease (CAD) was present in 63% overall and was more frequent in LGAS subtypes, particularly pLFLGAS (79.2%) and NFLGAS (72.7%), compared with HGAS (58.8%). NYHA class IV symptoms were most common in cLFLGAS (52.2%) and NFLGAS (45.5%). Risk scores varied across subtypes; median EuroSCORE was highest in cLFLGAS patients (6.91), with lower median values in HGAS, pLFLGAS, and NFLGAS (3.91, 3.48, and 3.27, respectively). STS scores followed a similar pattern (cLFLGAS median 5.00 vs. 3.05–3.60 in the other groups) (Table 1).

### 3.2. Differences in Calcium Quantification

Across hemodynamic AS subtypes, HGAS patients showed the highest median AVCS (3040 AU), indicating significantly greater valvular calcification compared to LGAS subtypes. pLFLGAS had the lowest AVCS (1.564 AU), while cLFLGAS (2399 AU) and NFLGAS (1873 AU) showed intermediate values. The interquartile ranges highlight substantial variability, particularly in pLFLGAS patients. The difference in AVCS across subtypes was statistically significant (*p* = 0.008). Similarly, CV was highest in HGAS patients (798 mm^3^) and progressively lower in cLFLGAS (644 mm^3^), NFLGAS (575 mm^3^), and pLFLGAS (448 mm^3^) patients. This difference was even more robust (*p* < 0.001), reinforcing the relationship between gradient severity and calcium burden in this TAVI cohort.

**Figure 3:**
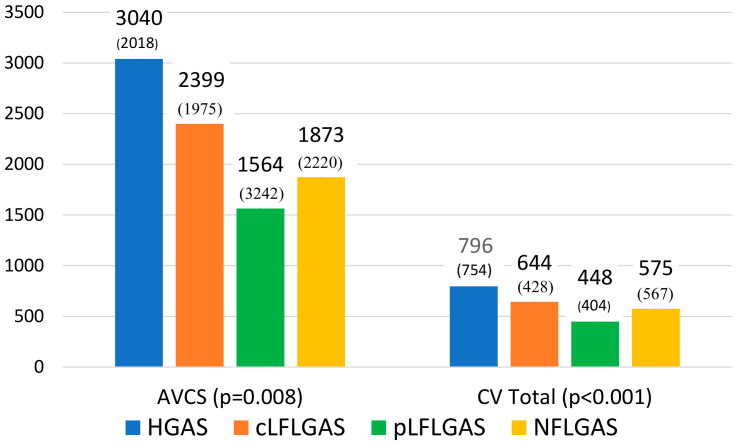
Aortic valve calcium score (AVCS) and calcium volume (CV) across the hemodynamic AS subtypes (median (Interquartile Ranges, IQR)).

**Figure 4:**
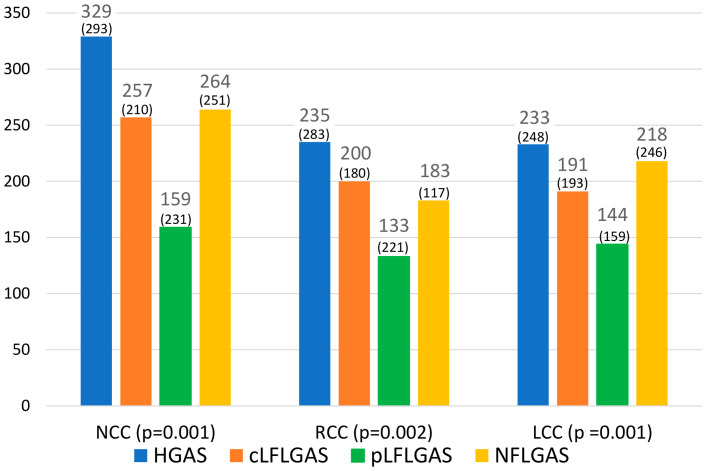
Calcium volume (CV) measured on contrast-enhanced CT across the hemodynamic AS subtypes in comparison to the different cusps (median (IQR)).

Median calcification was consistently highest in the HGAS group across all three cusps, with statistically significant differences observed between subtypes (Figure 3). The NCC, in particular, exhibited the highest overall calcification burden among all subtypes (Figure 4).

### 3.3. Mortality Outcomes and Periprocedural Complications

There were no statistically significant differences regarding all-cause mortality across AS subtypes at any observed time point (30 days, 1 year, 2 years after TAVI).

**Figure 5:**
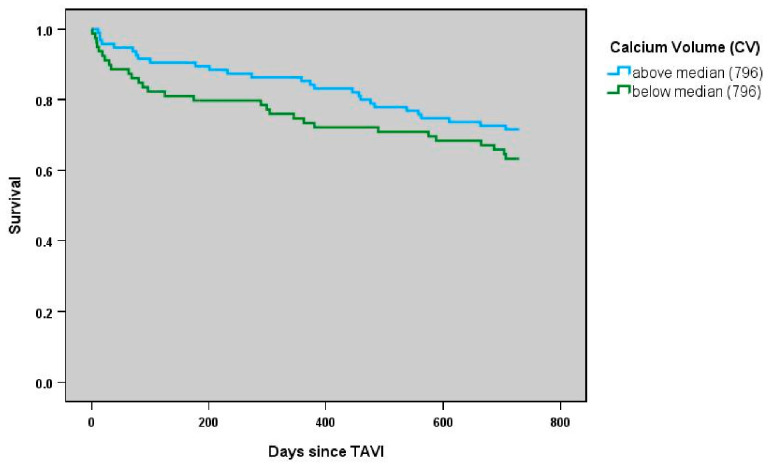
Kaplan–Meier survival curves comparing 2-year all-cause mortality in patients with calcium volume (CV) below vs. above the median.

The relationship between aortic valve calcification and mortality at 30 days, 1 year, and 2 years following TAVI was assessed by stratifying patients into two groups based on the median CV (group 1: ≥median; group 2: <median). At 30 days, mortality was 3.4% in the higher CV group and 7.5% in the lower CV group. The difference did not reach statistical significance (*p* = 0.24). At 1 year, mortality increased but remained numerically higher in patients with lower CV (22.8%) compared to those with higher CV (14.6%). However, this trend, again, did not reach statistical significance (*p* = 0.15). By 2 years, the gap narrowed slightly, with mortality rates of 28.4% in the high CV group and 36.7% in the low CV group. Although numerically higher in the low CV group, this difference was also not statistically significant (*p* = 0.26). Kaplan–Meier survival curves are shown in Figure 5.

**Figure 6:**
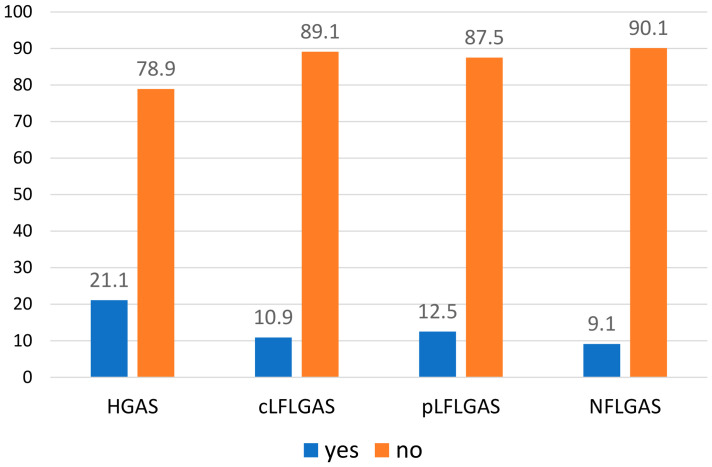
Thirty-day periprocedural complications (in %) across hemodynamic AS subtypes.

**Figure 7:**
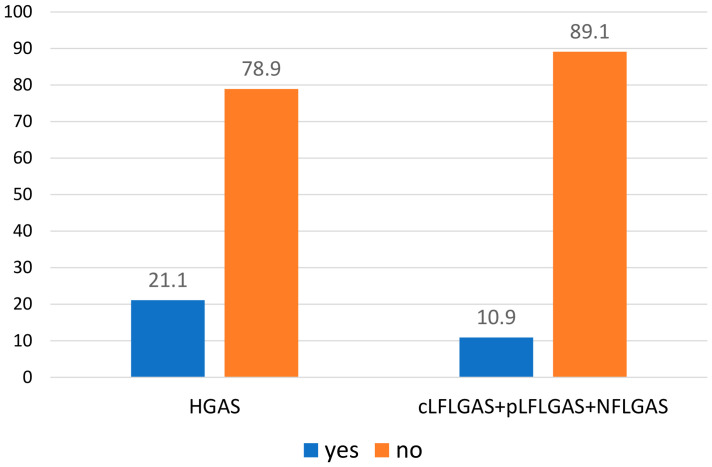
Thirty-day periprocedural complications (in %) comparing HGAS vs. pooled LGAS subtypes.

Among HGAS patients, 21.1% experienced at least one periprocedural complication, while the incidence was notably lower at 10.9% in the pooled LGAS subtypes group. Complications assessed in this analysis included vascular complications, overall bleeding events, bleeding requiring transfusion, pericardial tamponade, the need for permanent pacemaker implantation, and in-hospital mortality (Figure 6 and Figure 7, Table 2 and Table 3). Given the very small number of complications in some subgroups, differences should be interpreted with caution.

## 4. Discussion

The key finding of this retrospective single-center study is that patients with different LGAS subtypes exhibit lower aortic valve calcium levels compared to those with HGAS. When interpreting this result, one has to consider the relatively small sample size in some LGAS subtypes, in particular pLFLGAS and NFLGAS, which limits statistical power.

Secondly, CV was not associated with 2-year mortality, and survival rates were comparable between LGAS subtypes and the HGAS group.

In our cohort, patients with LGAS subtypes demonstrated significantly lower AVCS and CV values compared to HGAS, consistent with findings from previous studies [5,7,8]. The CT-derived AVCS thresholds differentiate accurately between moderate and severe AS in HGAS but appear less reliable in LGAS phenotypes, reinforcing the need for phenotype-specific diagnostic thresholds [8,9]. These findings are compatible with a less calcific and potentially more fibrotic remodeling pattern in LGAS subtypes; however, myocardial or valvular fibrosis was not directly assessed in this study. Several factors beyond stenosis severity may influence AVC burden, including sex and hemodynamic phenotype [10]. Veulemans, Piayda et al. (2021) reported that only in men, AVC levels were able to distinguish severe LGAS from moderate AS, suggesting a possible sex-specific diagnostic threshold [9]. In a dedicated analysis of 567 patients with severe tricuspid AS, asymmetrical calcification was predominant (78.1%), particularly affecting the non-coronary cusp (NCC) [6]. In our cohort, the NCC was more calcified than the other cusps. Consistent with these findings, Veulemans, Piayda et al. (2021) reported that the NCC is consistently the most heavily calcified cusp across all AS subtypes and sexes [9].

In this study, higher AVC was not associated with increased 2-year mortality, and survival outcomes were comparable between LGAS subtypes and the HGAS group. Regarding the missing association between calcium burden and survival observed by us, it cannot be stated clearly whether this is due to the limited power of the study or reflects a true absence of prognostic significance. While previous studies have reported conflicting results regarding the prognostic value of AVCS in LGAS subtype patients undergoing TAVI, our findings add to the growing evidence that calcification burden alone may not predict long-term outcomes in this population. Notably, despite the cLFLGAS group in this study presenting with higher surgical risk scores (EuroSCORE II and STS score) and more advanced symptoms, their mortality rates were comparable to those of other groups during follow-up. This suggests that patients in this high-risk subgroup may derive substantial benefit from TAVI, potentially offsetting their baseline risk profile. In a retrospective series of 526 patients, high AVC density was associated with improved survival following TAVI in cLFLGAS, but not in pLFLGAS [11]. Similarly, Aksoy, Cam et al. (2014) reported worse long-term outcomes among LGAS subtype patients with lower calcium scores [12]. However, Fischer-Rasokat, Renker et al. (2021) found that across 1.792 TAVI patients, including 650 with LGAS subtypes, AVC levels did not impact 1-year survival, indicating that the prognostic value of AVCS may be attenuated after valve replacement [13]. The CURRENT AS Registry-2 demonstrated that both cLFLGAS and pLFLGAS were associated with increased rates of death and heart failure hospitalization compared to HGAS, independent of valve calcium severity. On the contrary, outcomes in NFLGAS patients were similar to those in HGAS [14]. In line with our results, Juhász, Vecsey-Nagy et al. (2025) showed that a low AVCS is more prevalent in LGAS subtypes but does not predict the outcomes [15]. Furthermore, other studies showed that patients with higher CV showed lower mortality at 1 year after TAVI [16], which might reflect more benefit from TAVI in patients with higher calcification. The diagnosis of severe AS in patients with LGAS subtypes is complex, and the heterogeneity of this patient population, as well as the selection bias, likely contributes to the inconsistent findings reported across studies. Moreover, LGAS subtype patients are frequently excluded from randomized clinical trials. All conclusions drawn from the current study regarding pLFLGAS and NFLGAS patients are limited by the relatively low number of patients attributable to these subtype groups, which relevantly limit statistical power.

## 5. Limitations

This study has several limitations. First, it was conducted as a single-center retrospective analysis, which may limit generalizability, in particular external validity, and is subject to selection bias. Second, as is typical for retrospective cohorts, incomplete documentation led to occasional missing of clinical or imaging data, and in some cases, echocardiographic measurements were reconstructed retrospectively. Third, there was no core lab for imaging analysis, and earlier event adjudication had not been performed. Fourth, periprocedural endpoints were collected from routine clinical documentation rather than adjudicated in a dedicated events committee, and long-term outcomes beyond two years were not assessed. Fifth, subgroup sizes were modest, in particular for pLFLGAS and NFLGAS, reducing statistical power for outcome analyses. Sixth, AS severity in the LGAS subtype group was not proven by a uniform algorithm regarding additional methods like DSE and/or sex-specific CT-AVCS thresholds. Seventh, CV measurements on contrast-enhanced CT were performed using an older version of the 3mensio Structural Heart 10.7 SP1 software, which required manual adjustment of Hounsfield unit thresholds to identify calcification. This approach, while pragmatic, may have resulted in inter-operator variability and inconsistent detection of low-density calcifications, particularly in patients with lower calcium burden.

## 6. Conclusions

In this single-center retrospective tf-TAVI cohort, LGAS subtypes (cLFLGAS, pLFLGAS, and NFLGAS) accounted for approximately 30% of cases. Compared with HGAS, these phenotypes exhibited significantly lower aortic valve calcification burden, as measured by AVCS and CV, with a consistently higher cusp-specific burden in the NCC. In this single-center cohort, possibly underpowered for the LGAS subtypes pLFLGAS and NFLGAS, differences in valvular calcification were not associated with all-cause mortality up to 2 years after tf-TAVI.

## Figures and Tables

**Table 1:** Baseline characteristics across the HGAS, cLFLGAS, pLFLGAS, and NFLG patients.

	Total (*n* = 315)	HGAS (223; 70.8%)	cLFLGAS (46; 14.6%)	pLFLGAS (24; 7.6%)	NFLGAS (22; 7.0%)	*p*-Value (AS Subtypes)
Age (years)	81.4 ± 6.0	81.4 ± 6.0	81.5 ± 6.7	81.4 ± 6.2	81.9 ± 5.8	0.97
BMI (kg/m^2^)	27.3 ± 4.8	27.4 ± 4.9	27.1 ± 5.0	27.6 ± 4.1	26.8 ± 4.6	0.93
Female	159 (50.3%)	120 (53.8%)	18 (39.1%)	12 (50.0%)	9 (40.9%)	0.24
CAD	199 (63.0%)	131 (58.8%)	32 (69.6%)	19 (79.2%)	16 (72.7%)	0.014
NYHA IV	116 (36.7%)	74 (33.2%)	24 (52.2%)	8 (33.3%)	10 (45.5%)	
Euro-SCORE Median (min/max)	4.16 (0.8/38.5)	3.91 (0.8/34.8)	6.91 (1.9/38.5)	3.48 (1.2/13.9)	3.27 (1.5/10.5)	<0.001
STS-Score Median (min/max)	3.66 (1.0/27.6)	3.57 (1.0/27.6)	5.00 (1.3/20.5)	3.60 (1.3/23.8)	3.05 (1.6/9.0)	0.20

**Table 2:** Detailed periprocedural complications in patients with complications across hemodynamic AS subtypes (to report the percentage of complications, the total number of patients per subgroup is used as the denominator).

	Complications in All 57/315 pts.; 18.3%	Complications in HGAS 47/223 pts.; 21.1%	Complications in cLFLGAS 5/46 pts.; 10.9%	Complications in pLFLGAS 3/24 pts.; 12.5%	Complications in NFLGAS 2/22 pts.; 9.1%
Vascular complications	10/315 (3.2%)	6/223 (2.7%)	1/46 (2.2%)	2/24 (8.3%)	1/22 (4.5%)
Overall bleeding events	27/315 (8.6%)	19/223 (8.5%)	4/46 (1.84%)	3/24 (12.5%)	1/22 (4.5%)
Bleeding requiring transfusion	19/315 (6.0%)	12/223 (5.4%)	3/46 (6.5%)	3/24 (12.5%)	1/22(4.5%)
Pericardial tamponade	7/315 (2.2%)	6/223 (2.7%)	0/46 (0%)	0/24 (0%)	1/22 (4.5%)
Permanent pacemaker implantation	27/315 (8.6%)	23/223 (10.3%)	2/46 (4.3%)	2/24 (8.3%)	0/22 (0%)
In-hospital mortality	9/315 (2.9%)	7/223 (3.1%)	1/46 (2.2%)	1/24 (4.2%)	0/22 (0%)

**Table 3:** Detailed periprocedural complications in patients with complications comparing HGAS vs. pooled LGAS subtypes (to report the percentage of complications, the total number of patients per group is used as the denominator).

	Complications in All 57/315 pts.; 18.3%	Complications in HGAS 47/223 pts.; 21.1%	Complications in cLFLGAS + pLFLGAS + NFLGAS 10/92 pts.; 10.9%
Vascular complications	10/315 (3.2%)	6/223 (2.7%)	4/92 (4.3%)
Overall bleeding events	27/315 (8.6%)	19/223 (8.5%)	8/92 (8.7%)
Bleeding requiring transfusion	19/315 (6.0%)	12/223 (5.4%)	7/92 (7.6%)
Pericardial tamponade	7/315 (2.2%)	6/223 (2.7%)	1/92 (1.1%)
Permanent pacemaker implantation	27/315 (8.6%)	23/223 (10.3%)	4/92 (4.3%)
In-hospital mortality	9/5315 (2.9%)	7/223 (3.1%)	2/92 (2.2%)

## Data Availability

Data are included in this manuscript or available on request by the corresponding author.

## Abbreviations

AS	aortic stenosis
AVA	aortic valve area
AVC	aortic valve calcium
AVCS	aortic valve calcium score
AU	Agatston unit
CAD	coronary artery disease
CT	computed tomography
ceCT	contrast-enhanced CT
ncCT	non-contrast CT
CV	calcium volume
HU	Hounsfield units
LVEF	left ventricular ejection fraction
LVOT	left ventricular outflow tract
HGAS	high-gradient aortic stenosis
LG AS	low-gradient aortic stenosis
cLFLGAS	classical low-flow low-gradient aortic stenosis
pLFLGAS	paradoxical low-flow low-gradient aortic stenosis
NFLGAS	normal-flow low-gradient aortic stenosis
PG	pressure gradient
SVi	stroke volume index
LCC	left coronary cusp
NCC	non-coronary cusp
RCC	right coronary cusp
TAVI	transcatheter aortic valve implantation
TF-TAVI	transfemoral TAVI
VARC	Valve Academic Research Consortium
